# The Role of Iron and Other Micronutrients in Arterial Stiffness: Univariable and Multivariable Mendelian Randomization

**DOI:** 10.31083/RCM27920

**Published:** 2025-05-20

**Authors:** Weishen Qiao, Qi Liu, Hongyu Ding, Gang Wang, Yufei Sun, Zhibo Yao, Xingtao Huang, Xuedong Wang, Chao Fu, Jingbo Hou

**Affiliations:** ^1^Department of Cardiology Organization, The Second Affiliated Hospital of Harbin Medical University, 150001 Harbin, Heilongjiang, China; ^2^Key Laboratory of Myocardial Ischemia, Ministry of Education, Harbin Medical University, 150001 Harbin, Heilongjiang, China

**Keywords:** iron status, micronutrients, arterial stiffness, Mendelian randomization

## Abstract

**Background:**

Prior research on the relationship between iron status and arterial stiffness is limited, with causality still unclear. However, understanding these connections is crucial for improving the prevention and management of arterial stiffness. Therefore, this study aimed to examine the impact of iron status and other micronutrients on arterial stiffness risk using Mendelian randomization (MR) approaches.

**Methods:**

MR was performed utilizing genome-wide association studies (GWAS) data from European populations to investigate the causal link between various nutrients (iron, etc.) and arterial stiffness index. We selected the random-effects inverse-variance weighting (IVW) approach for the primary analysis and conducted numerous sensitivity tests to ensure accuracy.

**Results:**

This study found a causal effect of genetically predicted high levels of serum iron (β = 0.069, 95% confidence interval (CI) [0.031 to 0.107], *p*_FDR_ = 1.87 × 10^-3^) [false discovery rate, FDR], ferritin (β = 0.143, 95% CI [0.050 to 0.235], *p*_FDR_ = 8.28 × 10^-3^), and transferrin saturation (β = 0.053, 95% CI [0.025 to 0.080], *p*_FDR_ = 1.29 × 10^-3^) on arterial stiffness index. There was no evidence of reverse causality. Associations derived from multivariate MR analyses remained significant after adjusting for potential confounders. Zinc and carotene levels may be inversely linked with arterial stiffness.

**Conclusions:**

This study provides a genetic basis for the causal relationship between elevated iron status and increased arterial stiffness, suggesting the important role of micronutrients in the disease process.

## 1. Introduction

Arterial stiffness is a well-established risk factor for cardiovascular disease, characterized by degenerative changes in the extracellular matrix of blood vessel walls, which reduce vessel elasticity and impair their ability to stretch in response to changes in blood flow pressure [1]. Arterial stiffness is commonly evaluated using carotid–femoral pulse wave velocity (cfPWV) [2]. Additionally, the arterial stiffness index, a non-invasive parameter, is widely used to assess the severity of arterial stiffness [3]. An observational study found that patients with a higher arterial stiffness index exhibited at least a 25% increase in cardiovascular mortality and nearly a 40% rise in myocardial infarction rates over a mean follow-up period of three years [4]. Meanwhile, epidemiological studies have linked conditions such as obesity [5], diabetes [6], hypertension [7], and poor sleep patterns [8] to arterial stiffness. However, the causal relationship between micronutrients and arterial stiffness remains unclear.

Iron is an essential mineral nutrient in synthesizing and metabolizing various biological substances [9]. However, excessive iron can lead to the accumulation of reactive oxygen species (ROS), which are implicated in cardiovascular diseases [10, 11]. Recent studies have demonstrated an independent association between hyperferritinemia and arterial stiffness [12, 13]. Similarly, magnesium [14] and calcium [15] deficiencies have been linked to arterial stiffness, while zinc [16] and selenium [17] appear to exert protective effects by mitigating oxidative stress and inflammation in vascular walls. Nevertheless, current studies on micronutrients and arterial stiffness are mostly observational and cannot eliminate confounding or reverse causality, which limits our ability to draw conclusive causal inferences. In addition, existing studies lack comprehensive analyses of multiple micronutrients. Therefore, addressing this gap is essential to understand how micronutrients influence vascular health and develop more effective cardiovascular disease prevention strategies.

Mendelian randomization (MR) offers a powerful approach to infer causal relationships by using genetic variants as instrumental variables randomly assigned at conception. This method effectively eliminates confounding factors and ensures the correct direction of causality. Moreover, by leveraging data from genome-wide association studies (GWAS) [18], MR provides robust causal estimates across diverse populations. Thus, this study aimed to examine the possible causal link between genetically estimated iron indicators and genetical susceptibility to arterial stiffness employing bidirectional MR and multivariate MR (MVMR) analyses. Furthermore, we extended the study to investigate the associations of other minerals (calcium, magnesium, zinc, and selenium), vitamins (A, B6, B9, B12, C, D, and E), and carotene with arterial stiffness, offering a comprehensive perspective on the role of micronutrients in vascular health.

## 2. Materials and Methods

### 2.1 Study Design

**Fig. 1.**
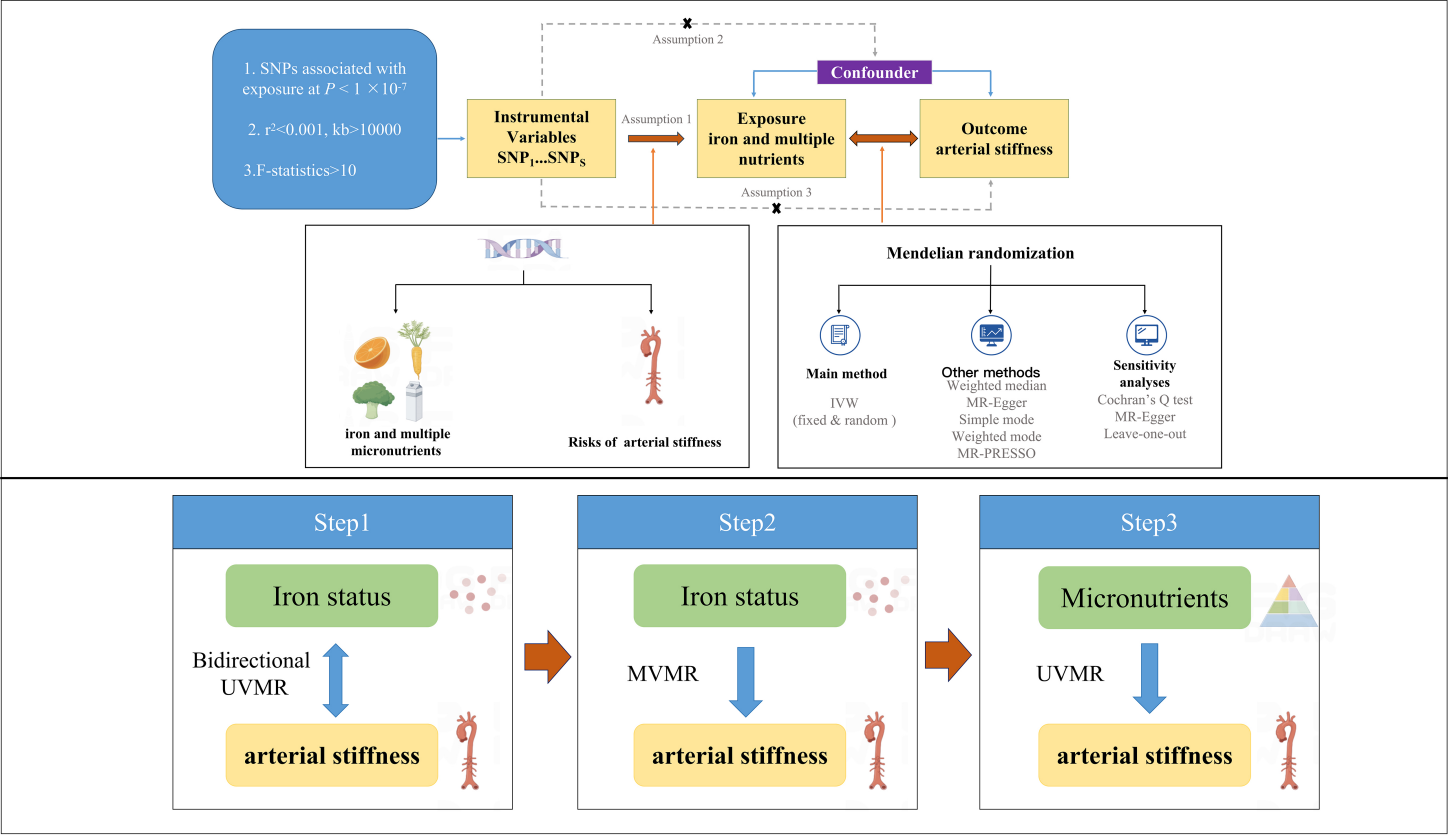
**Graphical overview of the MR study design**. MR, Mendelian randomization; UVMR, univariate Mendelian randomization; MVMR, multivariate Mendelian randomization; SNP, single nucleotide polymorphism; IVW, inverse-variance weighting; MR-PRESSO, Mendelian Randomization Pleiotropy Residual Sum and Outlier.

We first conducted univariate Mendelian randomization (UVMR) analyses to evaluate the potential causal relationship between iron indicators and arterial stiffness while testing for reverse causality. After accounting for other relevant risk factors, we further applied MVMR to assess the effect of iron indicators on arterial stiffness. Moreover, we explored the impact of various other micronutrients on arterial stiffness using UVMR. Fig. 1 illustrates the study design and three fundamental model assumptions of MR: (1) single nucleotide polymorphisms (SNPs) must be significantly associated with the exposure; (2) SNPs should not be related to any potential confounders; (3) SNPs can affect the occurrence of outcomes only through exposure factors rather than other pathways. Notably, this study used an open database previously approved by a relevant review board and did not require further ethical approval.

### 2.2 Instrument Selection

The pertinent SNPs attained the genome-wide significance threshold (*p* < 5 × 10^-7^). Part of the instrumental variables (IVs) used for the analysis were screened according to *p* < 5 × 10^-6^ because of insufficient SNPs of certain micronutrients, such as vitamins and carotene. To ensure there was no linkage disequilibrium (LD) between SNPs, we conducted a screening process by pruning SNPs within a 10,000 kb window and an r^2^ < 0.001 threshold [19, 20]. Additionally, to minimize pleiotropy, we used the LDlink online tool (https://ldlink.nih.gov/?tab=ldtrait) to verify whether the selected SNPs were unassociated with confounders and outcomes. F-statistics were performed for the selected SNPs to assess the power of each instrument. F = R^2^(N – K – 1)/[K(1 – R^2^)], where R^2^ represents the cumulative explained variance of the selected SNPs. K denotes the number of SNPs considered for analysis, while N represents the number of samples in the selected GWAS. When the value of F exceeds 10, it indicates a substantial connection with exposure, meaning the results are unaffected by weak instrumental bias [21]. The remaining SNPs were included in the MR study for further investigation.

### 2.3 Data Sources

GWAS summary statistics for the arterial stiffness index were obtained from Fung *et al*. [22], which included a cohort of 127,121 individuals of European descent. This study employed photoplethysmography, a non-invasive technique that uses infrared finger sensors to record digital blood volume waveforms. This automated method detects the digital volume pulse, characterized by a dicrotic waveform comprising direct and reflected components. The arterial stiffness index was calculated by dividing the standing height by the time interval between the peaks of the direct and reflected components, with higher values indicating increased arterial stiffness [23]. In addition, statistics were gathered for the pulse wave arterial stiffness index (n = 151,053) to perform further reliability validation. The Genetics of Iron Status Consortium (GIS) collected data on iron levels, gathering information on four key measures: serum iron, ferritin, transferrin, and transferrin saturation [24]. Data were gathered from 23,986 individuals with European heritage across 11 cohorts stationed at 9 participating centers. Other datasets were pooled to identify the causal effects of additional micronutrients on arterial stiffness. GWAS data for other crucial mineral elements contained calcium, magnesium (n = 64,979), zinc (n = 2603), and selenium (n = 2603), which were all extracted from individuals of European ancestry. Pooled data on varieties of vitamins (A, B6, B9, B12, C, D, and E) and carotene were acquired from the UKBiobank. Table 1 (Ref. [22, 25]) lists the details of the summary statistics. We selected these datasets for their large sample sizes, relevance to the studied micronutrients and outcomes, and proven quality in previous research. Their broad population representation enhances the generalizability of our findings.

**Table 1.** **Characteristics of the data source for exposure and outcomes**.

Traits	Consortium	Participants	Ancestry	GWAS ID
Iron	GIS	23,986 individuals	European	ieu-a-1049
Ferritin	GIS	23,986 individuals	European	ieu-a-1050
Transferrin	GIS	23,986 individuals	European	ieu-a-1052
Transferrin saturation	GIS	23,986 individuals	European	ieu-a-1051
Calcium	Neale lab	/	European	ukb-d-30680_irnt
Magnesium	MRC-IEU	64,979 individuals	European	ukb-b-7372
Zinc	Evans *et al*. [25]	2603 individuals	European	ieu-a-1079
Selenium	Evans *et al*. [25]	2603 individuals	European	ieu-a-1077
Vitamin A	MRC-IEU	62,991 individuals	European	ukb-b-17406
Vitamin B6	MRC-IEU	64,979 individuals	European	ukb-b-7864
Vitamin B9	MRC-IEU	460,351 individuals	European	ukb-b-3563
Vitamin B12	MRC-IEU	64,979 individuals	European	ukb-b-19524
Vitamin C	MRC-IEU	64,979 individuals	European	ukb-b-19390
Vitamin D	MRC-IEU	64,979 individuals	European	ukb-b-18593
Vitamin E	MRC-IEU	64,979 individuals	European	ukb-b-6888
Carotene	MRC-IEU	64,979 individuals	European	ukb-b-16202
Arterial stiffness index	Fung K *et al*. [22]	127,121 individuals	European	ebi-a-GCST008403
Pulse wave arterial stiffness index	MRC-IEU	151,053 individuals	European	ukb-b-11971

GWAS, genome-wide association studies; GIS, Genetics of Iron Status Consortium; MRC-IEU, MRC Integrative Epidemiology Unit.

### 2.4 Statistical Analysis

We harmonized the aggregated statistics to ensure that the alleles of each SNP were consistent between every nutrient and arterial stiffness. Several widely used MR analysis techniques were employed in this investigation to examine causal relationships. The random-effects inverse-variance weighting (IVW) method was selected as the primary approach, and it assumes that all genetic variants are valid instruments and that there is no evidence of pleiotropic effects [26]. Although significant effects of pleiotropy genetic variables may contribute to the low statistical power of MR-Egger, this analysis can infer modified causal effects and provide unbiased estimates even when some instruments are invalid [27]. The weighted median method can yield precise and strong effect estimates if a minimum of 50% of the data from reliable instruments are available [28]. Simple and weighted mode regressions were also applied as supplementary analyses.

Pleiotropy detection and correction were performed using MR-Egger regression [29], and further assessment of pleiotropy was conducted using the Mendelian Randomization Pleiotropy Residual Sum and Outlier (MR-PRESSO) test, which identifies and removes outlier SNPs associated with pleiotropy [30]. Heterogeneity was evaluated using Cochran’s Q test [28], with *p* > 0.05 indicating no heterogeneity and *p* < 0.05 suggesting the presence of heterogeneity. In cases where heterogeneity was detected (*p* < 0.05), the primary analysis was performed using the IVW method with a multiplicative random-effects model; otherwise, a fixed-effects model was used. We also conducted a leave-one-out sensitivity analysis to assess the robustness of the findings by removing each SNP individually and re-estimating the causal effects. If removing any SNP did not significantly affect the results, the credibility of the MR analysis was strengthened. Additionally, we used scatter plots, funnel plots, and forest plots to visualize results and evaluate causality at the individual SNP level.

Considering multiple testing corrections, we implemented false discovery rate (FDR) correction to adjust the *p*-values. We deemed a *p*_FDR_ below 0.05 in the UVMR analysis compelling evidence for a significant relationship, while those *p* < 0.05 but *p*_FDR_ > 0.05 were considered nominally significant. R software (Version 4.1.2; R Foundation for Statistical Computing, Vienna, Austria; https://www.r-project.org/) was utilized to conduct statistical analyses in collaboration with the “TwoSampleMR” and “MR-PRESSO” packages [31].

### 2.5 MVMR Analysis

MVMR, a further extension of MR, was applied to examine the direct causality between iron homeostasis and arterial stiffness. Genetic variants for potential confounders were obtained from the IEU Open-GWAS database (https://gwas.mrcieu.ac.uk). The analysis applied MVMR-IVW and the least absolute shrinkage and selection operator (LASSO) methods [32]. If at least one obtained a significant result, the causal relationship was considered to persist even after multivariate adjustment. To account for genetic confounding, characteristics such as obesity [33], hypertension [7], diabetes [6], smoking [34], alcohol consumption [35], and insomnia [36] were included in the multivariate analyses (**Supplementary Table 1**). Each confounder was added to the multivariate models separately and also adjusted independently for both the mothers of disease (obesity, hypertension, and diabetes) and the killers of health (smoking, alcohol, and insomnia).

## 3. Results

### 3.1 SNP Selection and Validation

Based on our pre-established inclusion criteria, we identified SNPs significantly associated with various iron status parameters or multiple micronutrients. These SNPs can serve as IVs for conducting the MR analysis. For more detailed information on F-statistics, please refer to** Supplementary Table 2**.

### 3.2 Iron Status and Arterial Stiffness

**Fig. 2.**
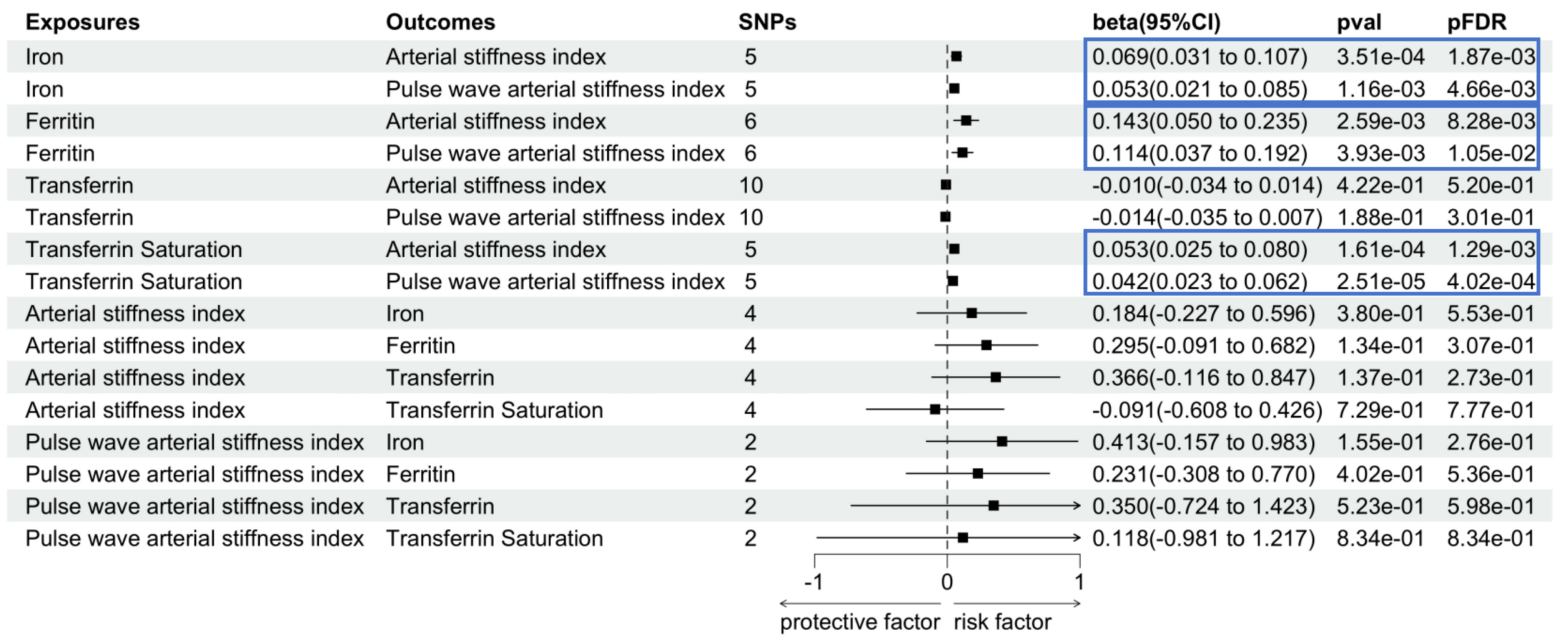
**Associations between genetically predicted iron status and arterial stiffness**. The blue box is used to emphasize key results, enhancing their visual distinction within the figure. SNP, single nucleotide polymorphism; CI, confidence interval; FDR, false discovery rate; val, value.

As illustrated in Fig. 2, the findings derived from the IVW analysis approach convincingly exhibited a substantial positive correlation between iron status and arterial stiffness. Researchers presented evidence of a significant connection between serum iron level and elevated arterial stiffness index (β = 0.069, 95% confidence interval (CI) [0.031 to 0.107], *p*_FDR_ = 1.87 × 10^-3^). This means that for every 1 SD increase in serum iron level, the arterial stiffness index increases by 0.069 units. It is suggested that the rise in serum iron may lead to arterial stiffness and increase the risk of cardiovascular disease. The confidence intervals further reinforce the reliability of these estimates, with all intervals excluding zero, confirming the robustness of the observed associations. Meanwhile, the estimates from the weighted median (β = 0.077, 95% CI [0.045 to 0.109], *p*_FDR_ = 1.21 × 10^-5^) and weighted mode regression (β = 0.078, 95% CI [0.047 to 0.110], *p*_FDR_ = 3.27 × 10^-2^) demonstrate that the causality evidence for serum iron level and arterial stiffness index is robust. MR-Egger regression, simple mode, and MR-PRESSO regression displayed the inconsistencies of the obtained risk estimates (**Supplementary Table 3**). In addition, our result discovered that the serum iron level had a significant causal effect on pulse wave arterial stiffness index (β = 0.053, 95% CI [0.021 to 0.085], *p*_FDR_ = 4.66 × 10^-3^). The weighted median showed similar estimates (**Supplementary Table 3**). We found evidence of a significant correlation between ferritin and arterial stiffness index (β = 0.143, 95% CI [0.050 to 0.235], *p*_FDR_ = 8.28 × 10^-3^). The weighted median regression exhibited similar estimates (β = 0.146, 95% CI [0.078 to 0.213], *p*_FDR_ = 6.90 × 10^-5^). We observed an association between ferritin and pulse wave arterial stiffness index (β = 0.114, 95% CI [0.037 to 0.192], *p*_FDR_ = 1.05 × 10^-2^). Meanwhile, weighted median regression also provided similar estimates (**Supplementary Table 3**). Evidence for the positive association between transferrin saturation and both arterial stiffness index and pulse wave arterial stiffness index was found in our study. For a 1 SD increment of genetically predicted transferrin saturation, the causal estimates increased by 0.053 (95% CI [0.025 to 0.080], *p*_FDR_ = 1.29 × 10^-3^) and 0.042 (95% CI [0.023 to 0.062], *p*_FDR_ = 4.02 × 10^-4^), respectively. Genetic liability to transferrin was not associated with either the arterial stiffness index or pulse wave arterial stiffness index. In addition, reverse causality validation was performed, and no obvious causality was found (**Supplementary Table 3**).

### 3.3 MVMR Results

In our further MVMR analysis, we found that after adjusting for obesity, hypertension, and diabetes, the associations between iron and arterial stiffness index remained generally strong (β_IVW_ = 0.060, 95% CI [0.028 to 0.092], *p*_IVW_ = 2.34 × 10^-4^), (β_LASSO_ = 0.060, 95% CI [0.028 to 0.092], *p*_LASSO_ = 2.34 × 10^-4^). Additionally, the associations were reliable after adjusting for smoking, alcohol, and insomnia (β_IVW_ = 0.075, 95% CI [0.046 to 0.105], *p*_IVW_ = 6.07 × 10^-7^), (β_LASSO_ = 0.081, 95% CI [0.053 to 0.110], *p*_LASSO_ = 2.12 × 10^-8^). Even after adjusting for all six confounders, the associations between iron and arterial stiffness index were also robust (β_IVW_ = 0.058, 95% CI [0.029 to 0.088], *p*_IVW_ = 8.06 × 10^-5^), (β_LASSO_ = 0.058, 95% CI [0.029 to 0.088], *p*_LASSO_ = 8.06 × 10^-5^) (**Supplementary Table 4**).

Furthermore, the associations between genetically determined levels of ferritin (β_IVW_ = 0.072, 95% CI [0.024 to 0.120], *p*_IVW_ = 3.41 × 10^-3^), (β_LASSO_ = 0.087, 95% CI [0.031 to 0.142], *p*_LASSO_ = 2.43 × 10^-3^) (**Supplementary Table 5**) and transferrin saturation (β_IVW_ = 0.050, 95% CI [0.027 to 0.073], *p*_IVW_ = 2.37 × 10^-5^), (β_LASSO_ = 0.050, 95% CI [0.027 to 0.073], *p*_LASSO_ = 2.37 × 10^-5^) (**Supplementary Table 6**) and arterial stiffness index were also robust after adjusting for all confounders.

### 3.4 Other Micronutrients and Arterial Stiffness

**Fig. 3.**
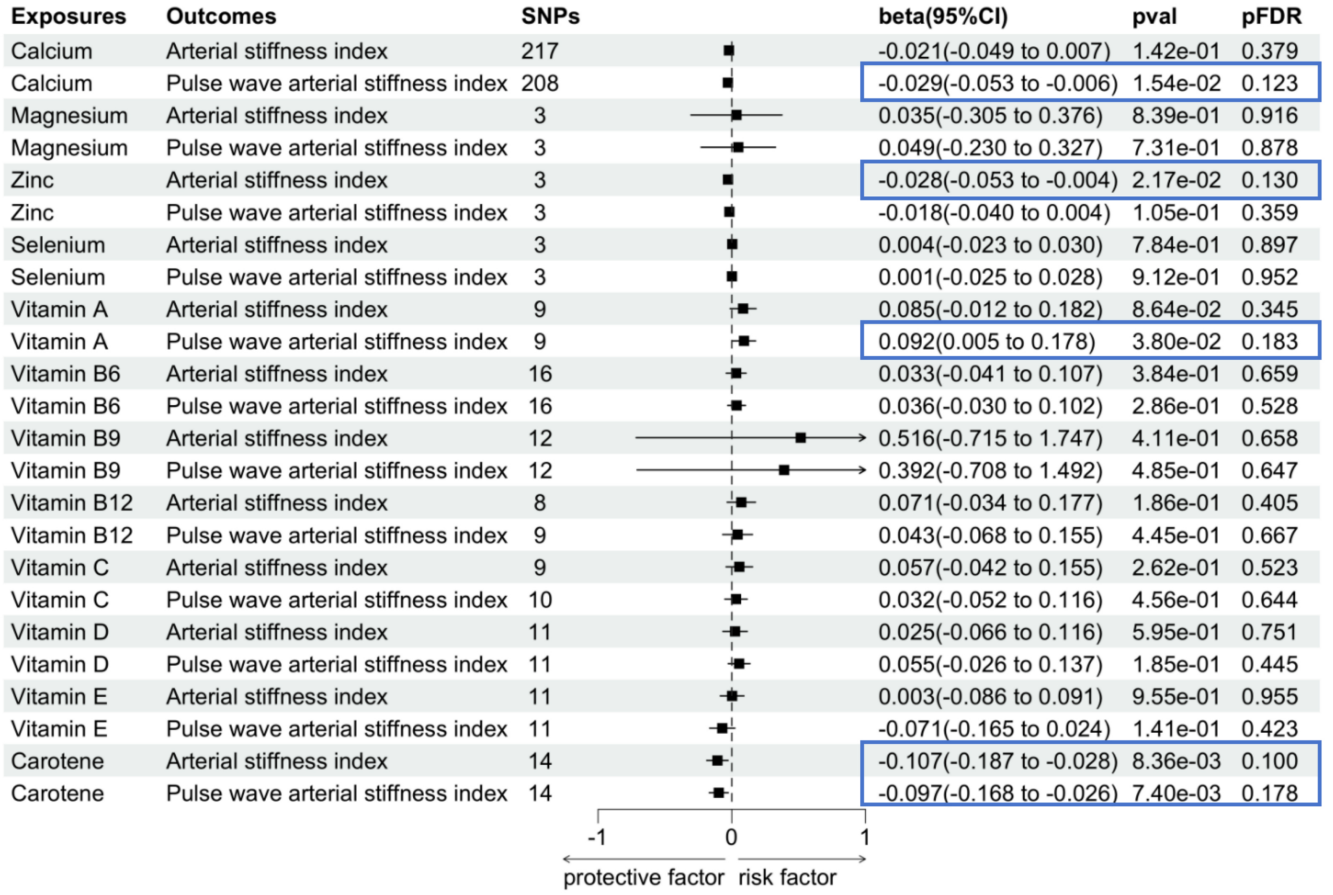
**Associations between genetically predicted other micronutrients and arterial stiffness**. The blue box is used to emphasize key results, enhancing their visual distinction within the figure. SNP, single nucleotide polymorphism; CI, confidence interval; FDR, false discovery rate; val, value.

In addition to iron status, we explored the causal link between several other mineral trace elements and arterial stiffness index by conducting UVMR (Fig. 3). Genetic evidence for higher zinc levels was linked to a decreased arterial stiffness index (β = –0.028, 95% CI [–0.053 to –0.004], *p* = 2.17 × 10^-2^, *p*_FDR_ = 1.30 × 10^-1^); however, this effect was suggested as not statistically significant. Moreover, no association was detected with pulse wave arterial stiffness index (β = –0.018, 95% CI [–0.040 to 0.004], *p* = 1.05 × 10^-1^, *p*_FDR_ = 3.59 × 10^-1^) (**Supplementary Table 3**). Meanwhile, the genetically predicted calcium level was associated with a decreased pulse wave arterial stiffness index (β = –0.029, 95% CI [–0.053 to –0.006], *p* = 1.54 × 10^-2^, *p*_FDR_ = 1.23 × 10^-1^). Conversely, vitamin A was associated with an increased pulse wave arterial stiffness index (β = 0.092, 95% CI [0.005 to 0.178], *p* = 3.80 × 10^-2^, *p*_FDR_ = 1.83 × 10^-1^). In contrast, no associations were observed for magnesium and selenium (**Supplementary Table 3**). We also detected carotene had a positive effect on arterial stiffness index (β = –0.107, 95% CI [–0.187 to –0.028], *p* = 8.36 × 10^-3^, *p*_FDR_ = 1.00 × 10^-1^) and pulse wave arterial stiffness index (β = –0.097, 95% CI [–0.168 to –0.026], *p* = 7.40 × 10^-3^, *p*_FDR_ = 1.78 × 10^-1^), indicating the important role of carotene supplementation in preventing and treating arterial stiffness. Surprisingly, vitamins B, C, D, and E demonstrated no obvious causal effect on arterial stiffness in the UVMR analysis (**Supplementary Table 3**).

### 3.5 Sensitivity Analysis

Sensitivity analysis was conducted to assess the dependability of the findings. Our Egger regression analysis showed no evidence of pleiotropy (*p* > 0.05). However, Cochran’s Q statistic revealed heterogeneity for certain outcomes, potentially due to sex and age differences among the population (**Supplementary Table 7**). MR-Egger analysis showed no evidence of pleiotropy. The results remained unchanged (*p*_distortion_ > 0.05) after excluding potential outliers using the MR-PRESSO method (**Supplementary Table 7**).

In the leave-one-out analysis, the single removal of any SNP did not significantly change the overall effect, indicating that no specific SNP significantly biased the MR analysis results. The scatter plots show the impact of SNPs on exposure and outcomes in our study and assess the direction and strength of causal relationships. The funnel plot is symmetrical, reflecting the uniform data distribution, which enhances the reliability and robustness of the research results (**Supplementary Figs. 1–9**).

## 4. Discussion

Our research aimed to explore the potential correlation between iron levels and arterial stiffness using bidirectional UVMR and MVMR analysis methods. The findings indicated that iron status may significantly affect arterial stiffness, with no evidence supporting inverse causality. Additional MVMR analyses demonstrated that the causal relationships between serum iron and the arterial stiffness index remained robust even after adjusting for six potential confounding variables. This suggests that iron status may be an independent risk factor for arterial stiffness. Furthermore, we provided potential evidence of a positive effect of elevated levels of zinc and carotene on arterial stiffness. These findings contribute to understanding the real-world implications of micronutrients on arterial health and support the potential for targeted nutritional or clinical interventions to reduce arterial stiffness.

Iron status, especially ferritin, is an independent predictor of various clinical outcomes and a marker of disease progression. Sciacqua *et al*. [13] highlighted that ferritin could independently predict arterial stiffness, consistent with our MR analysis. While Sciacqua *et al*. [13] focused on hypertensive patients, especially in the context of glucose tolerance, our study expands on this by investigating a broader population using GWAS data. The referenced research highlighted the interaction between ferritin, inflammation, and glucose tolerance in hypertensive patients. Similarly, we considered the broader biological implications of ferritin and adjusted for potential confounders using MVMR methods. Our results confirm that iron indicators, such as ferritin, are independent risk factors for arterial stiffness. In a study involving a population of 2932 Koreans [37], ferritin was also shown to be independently associated with arterial stiffness. Valenti *et al*. [12] reported that elevated serum ferritin levels were linked to an increased likelihood of arterial stiffness. In addition, increased common carotid intima-media thickness and the presence of carotid plaques were found to correlate with serum ferritin levels in patients undergoing hemodialysis and those with nonalcoholic fatty liver disease [38, 39]. Di Marco *et al*. [40] noted elevated inflammatory markers and ferritin in prediabetic patients, indicating significant changes in arterial stiffness and thickness parameters in this group.

The body can acquire iron through dietary intake and iron recycling within red blood cells. However, iron overload can produce ROS through the Fenton reaction [41], resulting in oxidative stress and promoting arterial stiffness development [42]. Moreover, iron status is associated with inflammatory factors, as inflammation can affect the arterial media and increase arterial stiffness [43]. The most extensive examination of cfPWV to date revealed a correlation between cfPWV and interleukin-6 (IL-6) levels in the Framingham cohort [44]. Additionally, the IL-12 and IL-18 levels have been shown to contribute to increased arterial stiffness in adults with a low cardiovascular risk [42, 45].

Serum zinc is involved in inhibiting vascular smooth muscle calcification, and an association has been reported between zinc deficiency and arterial stiffness [46]. Our MR analysis yielded a similar conclusion. Zinc is key in reducing oxidative stress by stabilizing cell membranes and regulating superoxide dismutase activity, an essential enzyme in mitigating ROS [47]. Additionally, zinc inhibits nuclear factor-kappa B activation, a critical pathway in vascular inflammation [48]. These mechanisms help maintain vascular elasticity and prevent endothelial dysfunction. Calcium concentration is related to the degree of arterial stiffness [49, 50], but our analysis showed a positive effect of calcium intake on arterial stiffness. Magnesium, a calcium channel blocker, is thought to improve vascular calcification [51, 52]; however, a randomized controlled trial by Schutten and co-authors [53] found no evidence of reduced arterial stiffness after 24 weeks. Additionally, magnesium supplementation has been shown to have varying effects on arterial stiffness depending on the form of magnesium administered (e.g., magnesium citrate vs. magnesium oxide vs. magnesium sulfate), the dosage, and the baseline characteristics of the participants. In addition, magnesium’s influence on arterial stiffness might not be linear. Selenium is an exogenous antioxidant nutrient, and selenium supplementation can improve elastin degradation in the vascular wall and reduce arterial stiffness [17, 54]. However, no causal relationship was established in our analysis. The relatively small sample sizes in the GWAS datasets for magnesium and selenium could have limited the statistical power of the MR analyses, making it challenging to detect weaker causal effects. Second, the genetic instruments for these micronutrients, while meeting the necessary strength and relevance criteria, may not fully capture the variance in their circulating levels, further reducing the sensitivity of our analysis. It is also possible that magnesium and selenium do not directly influence arterial stiffness, with prior findings potentially reflecting confounding or indirect pathways. Hence, future studies should utilize larger GWAS datasets and improved genetic proxies to enhance the robustness of MR analyses. In several studies, vitamins C, D, and E were shown to have a protective effect on arterial stiffness [55, 56, 57]. However, this was not observed in our study, potentially due to the impact not being linear.

Carotene, renowned for its remarkable anti-inflammatory and anti-oxidative properties, has been extensively researched and utilized across diverse domains, but controversy persists regarding its impact on arterial stiffness. A randomized controlled clinical trial reported no significant antioxidant effect after 12 weeks of treatment with antioxidants such as carotene [58]. Conversely, a meta-analysis by Ashor *et al*. [59] showed that antioxidant supplementation was associated with reduced arterial stiffness. Mechanistically, carotene scavenges free radicals, suppresses proinflammatory cytokines, such as IL-6 and tumor necrosis factor (TNF)-α [60], and improves endothelial function by enhancing nitric oxide bioavailability [61].

Our research has the following advantages. It was the first comprehensive and extensive MR study to prove the causality between micronutrients and arterial stiffness from a genetic perspective, addressing limitations inherent in traditional epidemiological research. MR offers significant advantages over conventional observational studies by addressing confounding factors and reverse causality. Using genetic variants as instrumental variables, MR minimizes the influence of lifestyle or environmental factors, providing unbiased estimates. For instance, in our study, genetic variants for iron-related biomarkers, independent of environmental or lifestyle factors, confirmed a causal relationship with arterial stiffness. Additionally, since genetic variants are fixed at birth, MR ensures the correct direction of causality, ruling out reverse effects. Compared to the inconsistencies often seen in observational studies, MR uses genetic data from large-scale GWAS, providing robust evidence supported by sensitivity analyses and reinforcing its reliability. Moreover, an additional validation dataset was employed to enhance the robustness of our findings; the presence of consistent outcomes significantly minimizes the likelihood of chance discoveries.

Our study also has several limitations that should be considered. First, there may be a slight overlap in participants within the datasets used for analysis, which may have introduced a small deviation. Future studies should prioritize the use of independent datasets from different databases. Researchers should ensure that genetic instruments are selected from well-curated and non-overlapping datasets. When using summary-level GWAS data, we suggest using other sources to cross-check results and minimize overlap bias. In our analysis, the relatively high F-statistics of the IVs minimized this bias. Additionally, applying techniques, such as leave-one-study-out analyses, can help identify and mitigate potential biases caused by data overlap. We also performed secondary MR analyses using entirely separate datasets for validation, which confirmed the consistency of our findings. Second, IVW was chosen as the primary analysis method due to its efficiency and simplicity, assuming that genetic instruments are valid and do not violate the assumption of no pleiotropy. However, we cannot completely rule out its potential influence, though our results did not provide evidence of horizontal pleiotropy based on the MR-Egger regression and MR-PRESSO tests. Our findings are consistent across different sensitivity analyses; the possibility of pleiotropic effects remains a limitation that should be considered when interpreting the results. Third, the GWAS data utilized in this study predominantly involved individuals of European ancestry. While this helped minimize population stratification bias, it may limit the generalizability of the findings to populations with different ancestral backgrounds. In addition, we failed to conclude the long-term effect of nutrient levels on arterial stiffness over time due to the cross-sectional nature of the GWAS data. Future research should aim to incorporate data from more diverse ethnic groups and longitudinal cohorts with repeated measures of nutritional levels and arterial stiffness to broaden the applicability. In addition, future studies incorporating functional genetics studies, such as transcriptomics and proteomics, can provide validation of the underlying molecular mechanisms. Integrating other omics approaches, such as metabolomics, could shed light on the intricate role of micronutrients in arterial stiffness, offering a more comprehensive understanding of the mechanisms involved.

## 5. Conclusions

Our research comprehensively explains the causal relationship between micronutrients and arterial stiffness, focusing particularly on key iron status indicators. Our study suggests elevated iron status, characterized by higher serum iron, ferritin, and transferrin saturation levels, is causally associated with increased arterial stiffness. Clinically, our results highlight the importance of strategies to monitor and manage iron levels, especially in populations at increased risk of arterial stiffness. Regular screening of iron status could be integrated into cardiovascular health assessments. Furthermore, promoting carotene-rich foods (e.g., carrots, leafy greens) and zinc-rich sources (e.g., nuts, seafood) in dietary guidelines or supplements could support vascular health. Further research is needed to refine these strategies and assess long-term benefits.

## Availability of Data and Materials

The datasets analyzed during the current study are available in the IEU OpenGWAS project, https://gwas.mrcieu.ac.uk.

## Supplementary Material

Supplementary material associated with this article can be found, in the online version, at https://doi.org/10.31083/RCM27920.
