# Supplementary material for: The Role of Iron and Other Micronutrients in Arterial Stiffness: Univariable and Multivariable Mendelian Randomization
Source: Rev Cardiovasc Med. 2025 May 20;26(5):27920. doi: 10.31083/RCM27920 (PMC12135662; doi:10.31083/RCM27920)
Supplement: Supplementary file 1 [file 2153-8174-26-5-27920-s1.zip › Supplementary Figure.pdf]

Supplementary Figures

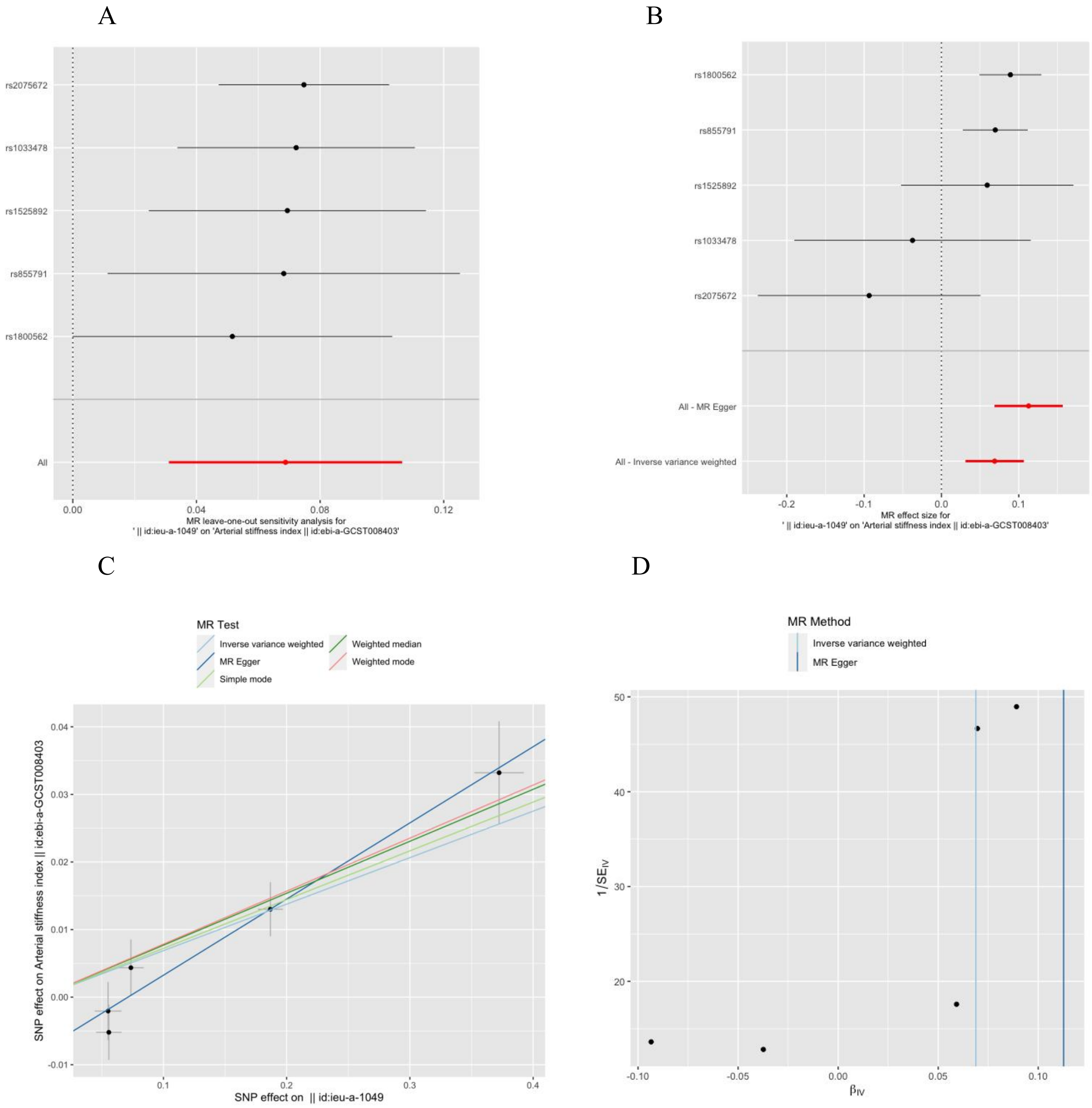

Supplementary Figure S1 Relationship between iron and arterial stiffness index is displayed in (A) a forest plot, (B) a leave-one-out sensitivity analysis, (C) a scatter plot, and (D) a funnel plot.

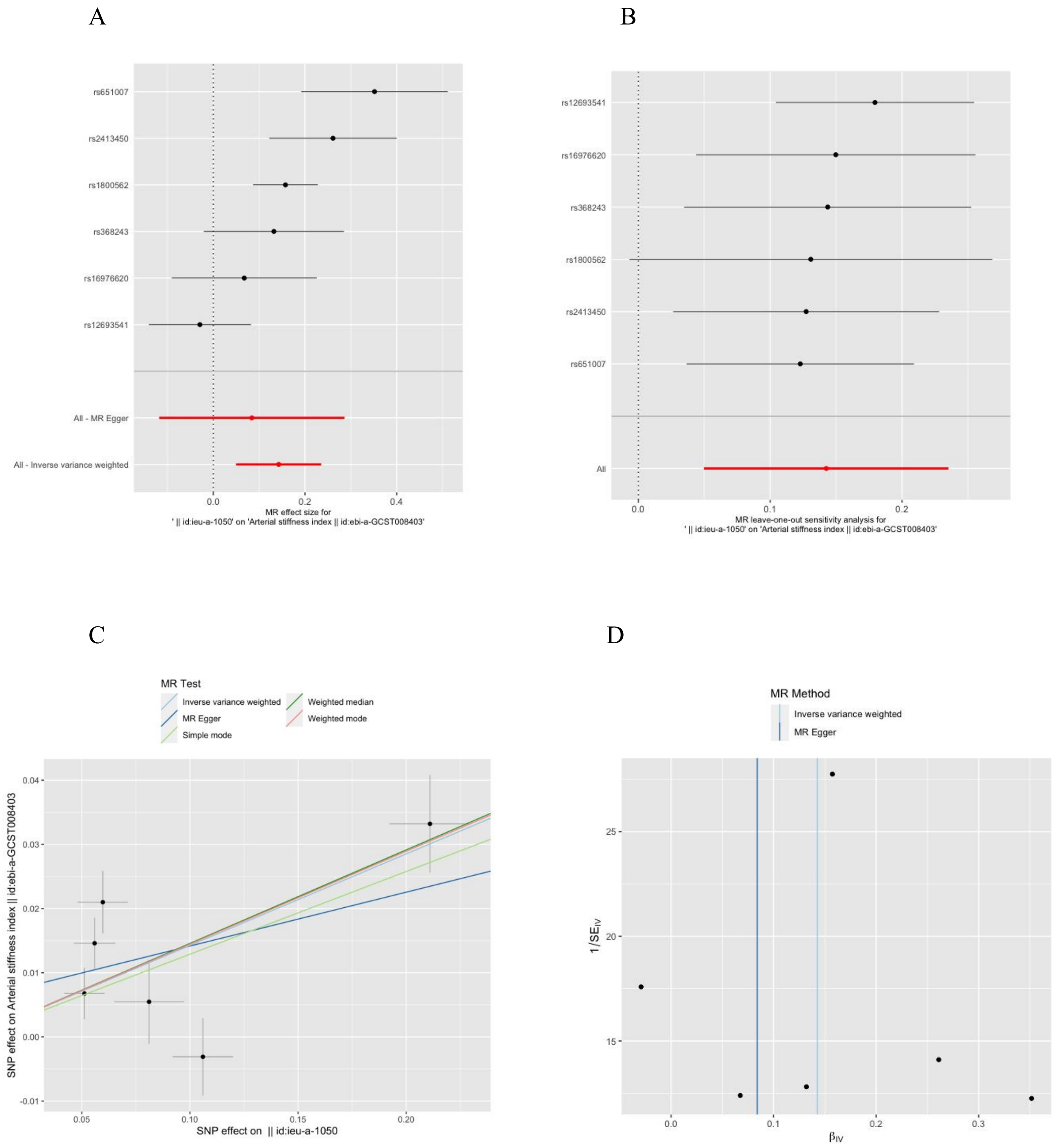

Supplementary Figure S2 Relationship between ferritin and arterial stiffness index is displayed in (A) a forest plot, (B) a leave-one-out sensitivity analysis, (C) a scatter plot, and (D) a funnel plot.

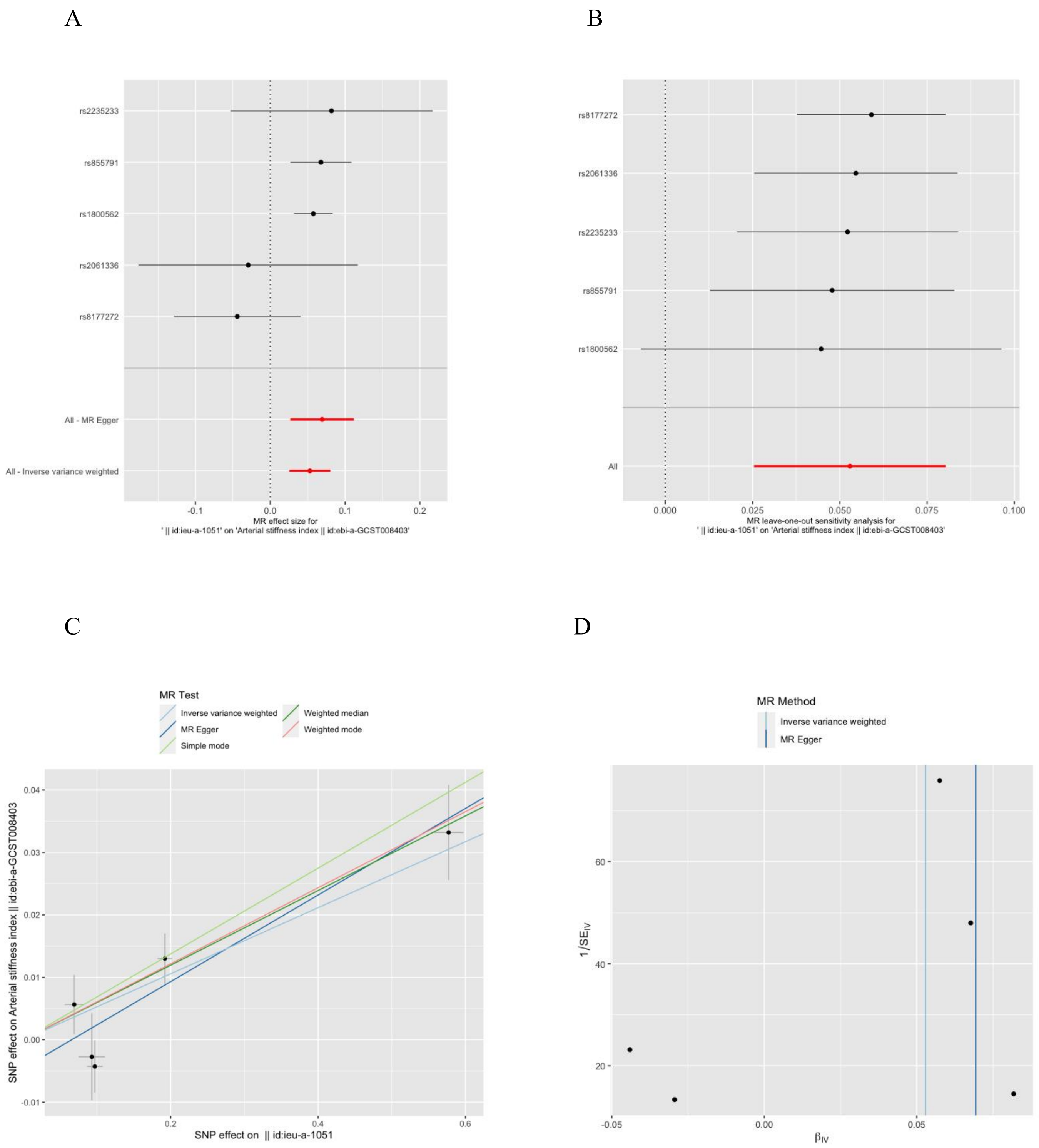

Supplementary Figure S3 Relationship between transferrin saturation and arterial stiffness index is displayed in (A) a forest plot, (B) a leave-one-out sensitivity analysis, (C) a scatter plot, and (D) a funnel plot.

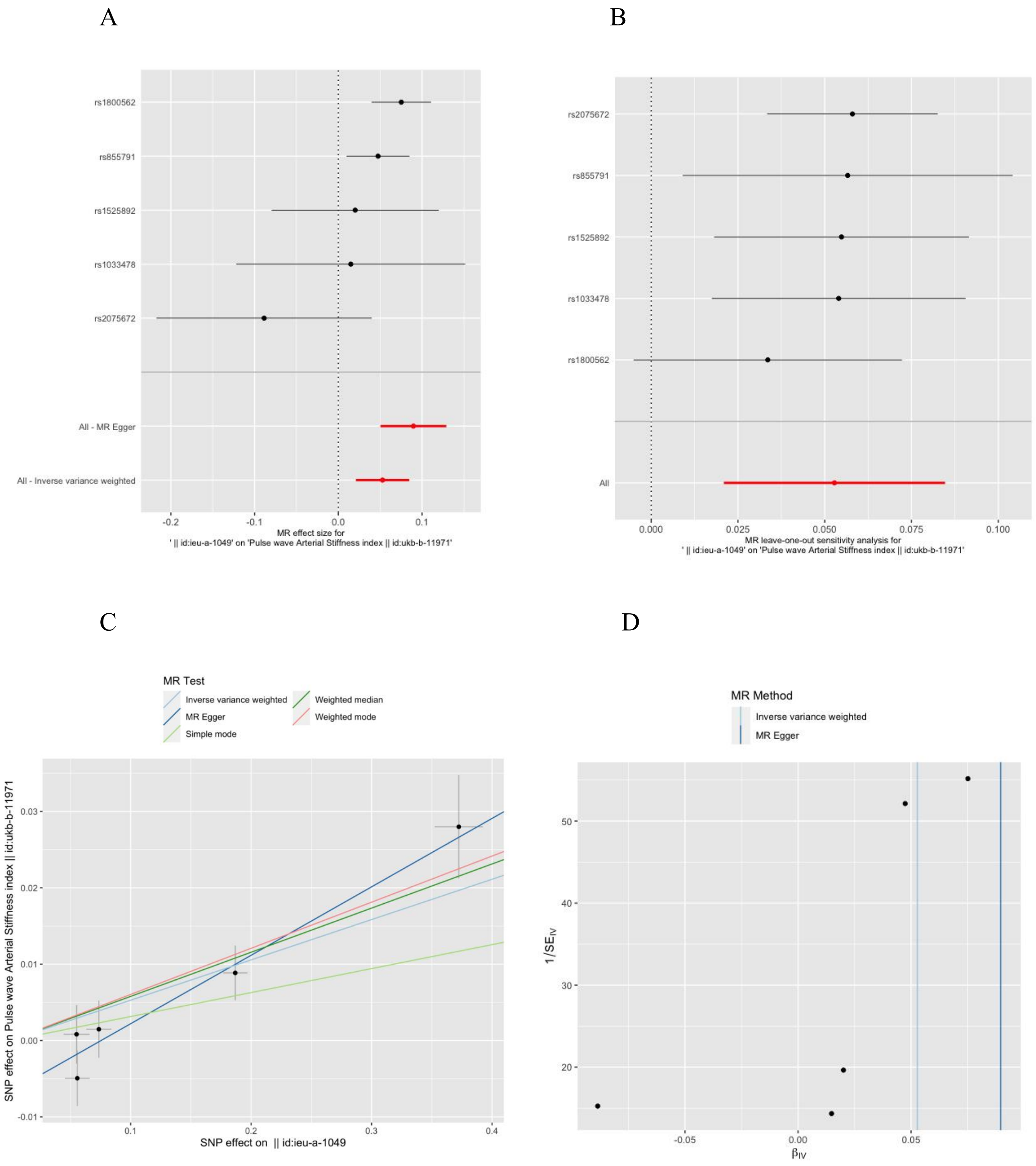

Supplementary Figure S4 Relationship between iron and pulse wave arterial stiffness index is displayed in (A) a forest plot, (B) a leave-one-out sensitivity analysis, (C) a scatter plot, and (D) a funnel plot.

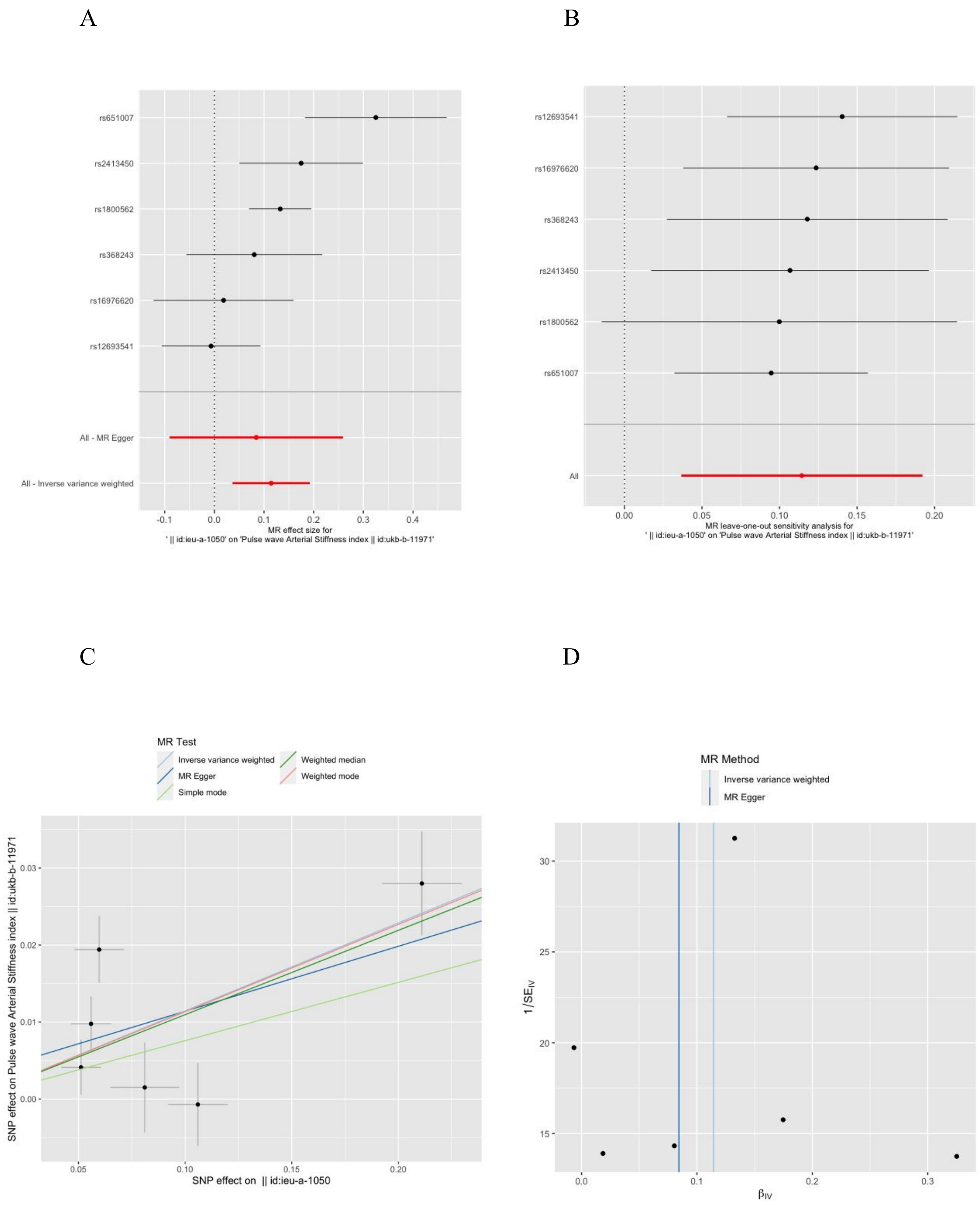

Supplementary Figure S5 Relationship between ferritin and pulse wave arterial stiffness index is displayed in (A) a forest plot, (B) a leave-one-out sensitivity analysis, (C) a scatter plot, and (D) a funnel plot.

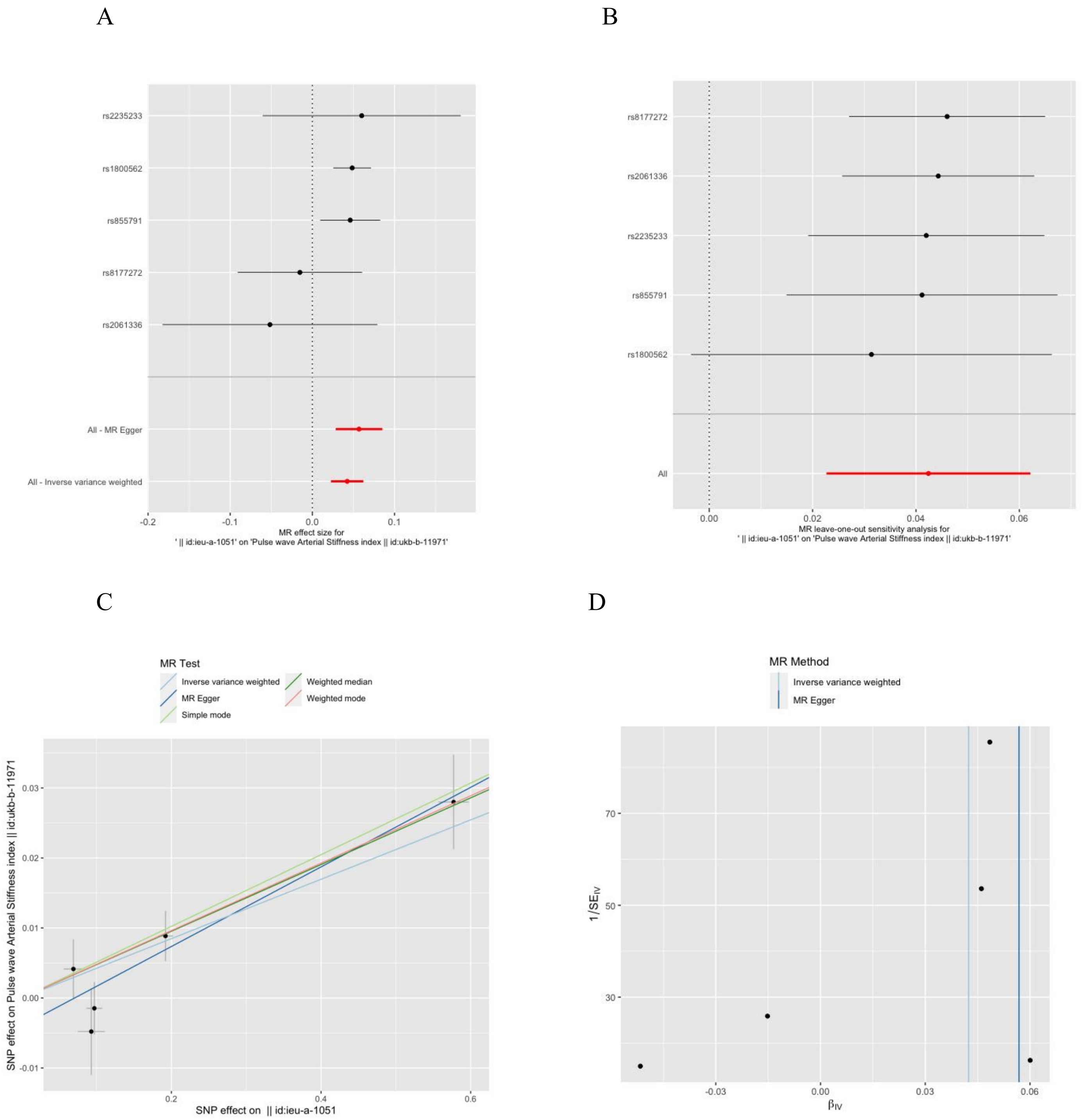

Supplementary Figure S6 Relationship between transferrin saturation and pulse wave arterial stiffness index is displayed in (A) a forest plot, (B) a leave-one-out sensitivity analysis, (C) a scatter plot, and (D) a funnel plot.

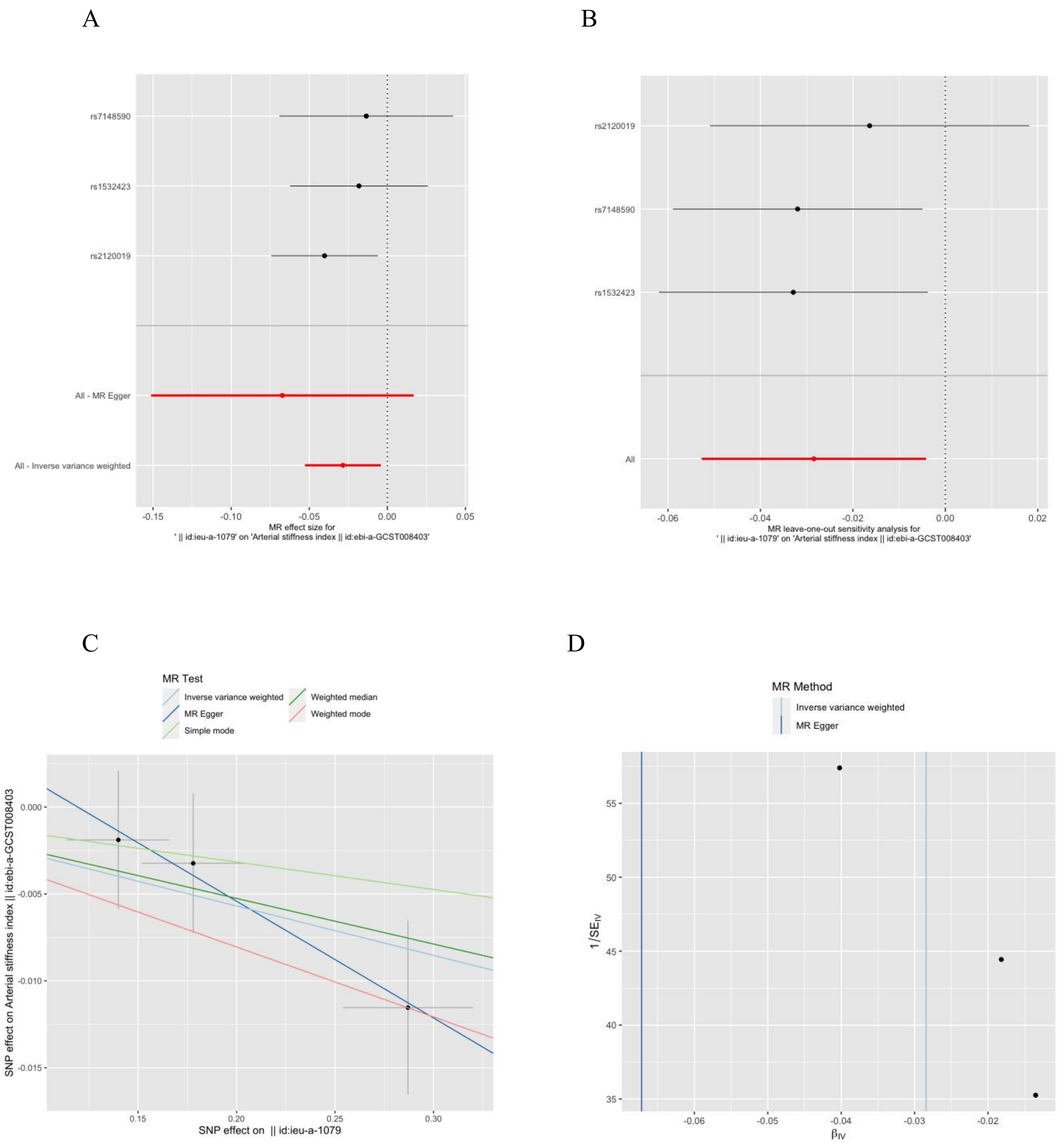

Supplementary Figure S7 Relationship between zinc and arterial stiffness index is displayed in (A) a forest plot, (B) a leave-one-out sensitivity analysis, (C) a scatter plot, and (D) a funnel plot.

A

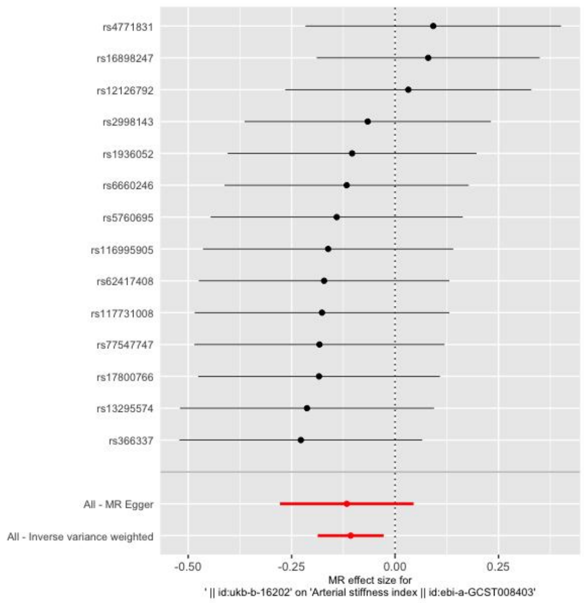

B

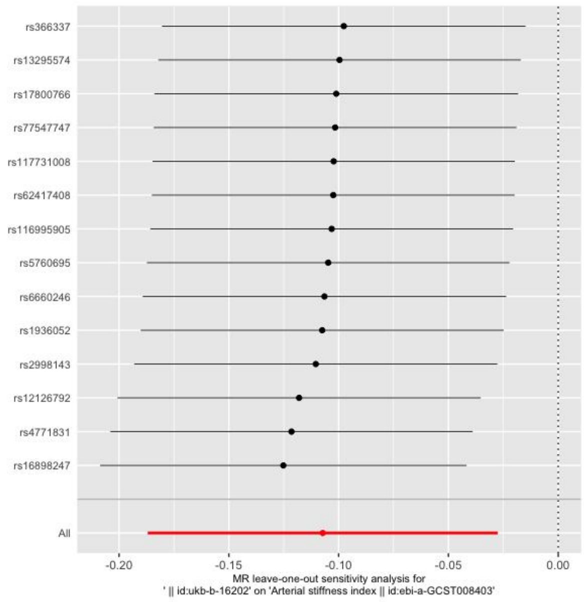

C

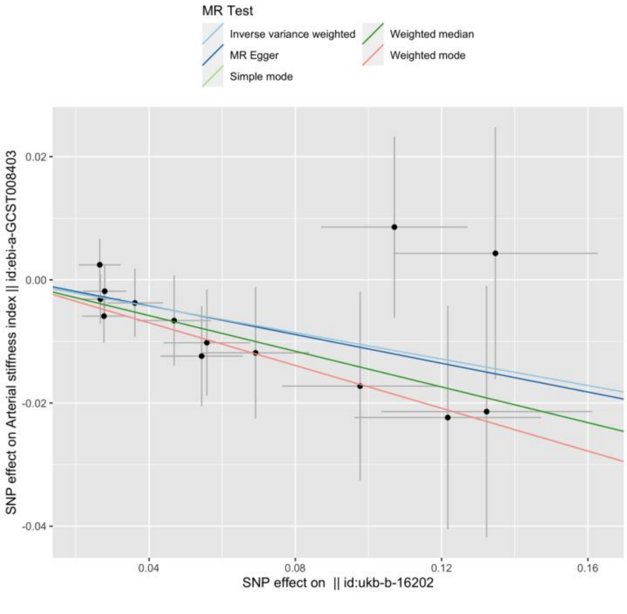

D

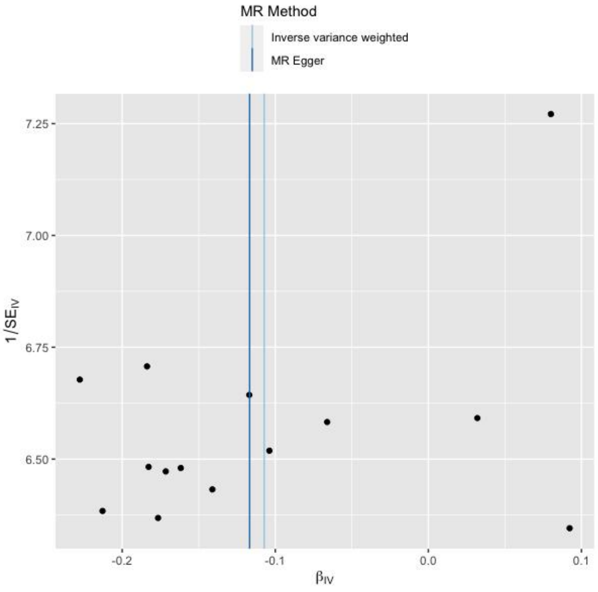

Supplementary Figure S8 Relationship between carotene and arterial stiffness index is displayed in (A) a forest plot, (B) a leave-one-out sensitivity analysis, (C) a scatter plot, and (D) a funnel plot.

A

B

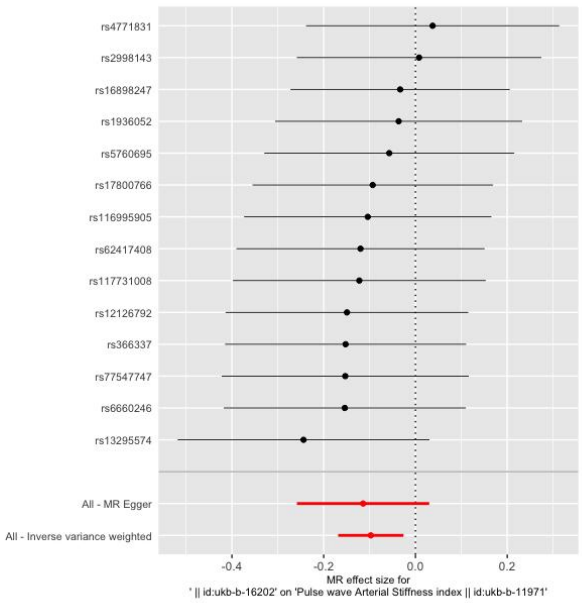

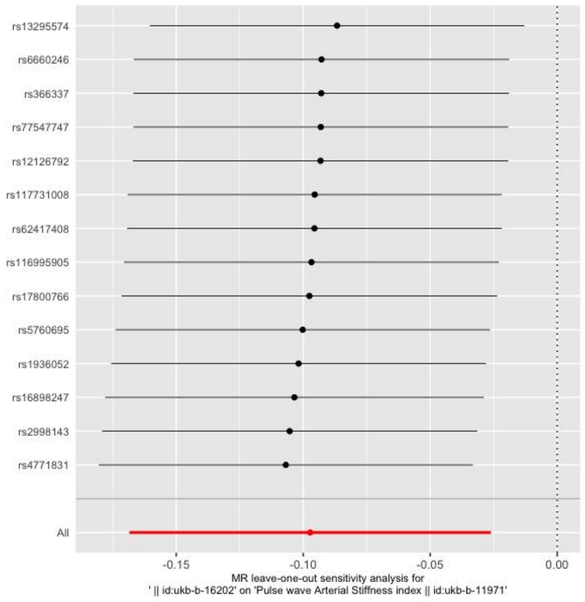

C

D

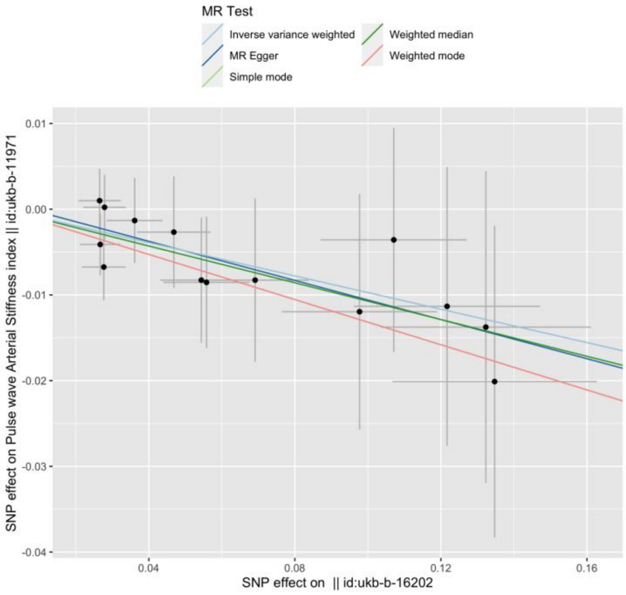

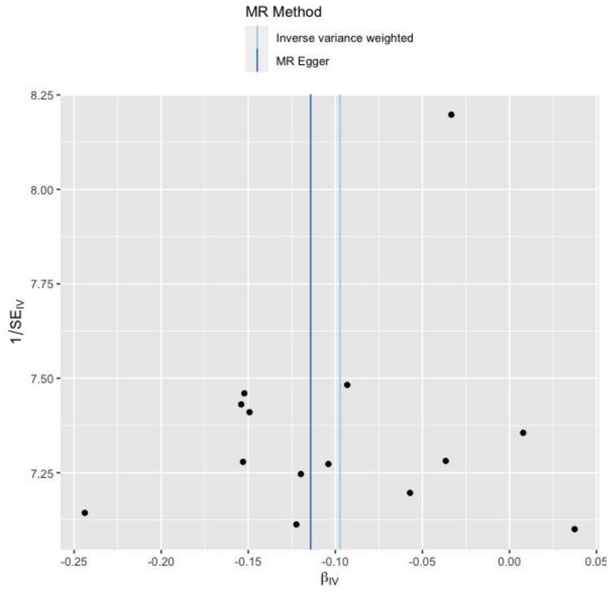

Supplementary Figure S9 Relationship between carotene and pulse wave arterial stiffness index is displayed in (A) a forest plot, (B) a leave-one-out sensitivity analysis, (C) a scatter plot, and (D) a funnel plot.
